# Identifying vital nodes based on reverse greedy method

**DOI:** 10.1038/s41598-020-61722-8

**Published:** 2020-03-16

**Authors:** Tao Ren, Zhe Li, Yi Qi, Yixin Zhang, Simiao Liu, Yanjie Xu, Tao Zhou

**Affiliations:** 1 Software College, Northeastern University of China, Shenyang, 110819 P. R. China; 2 CompleX Lab, University of Electronic Science and Technology of China, Chengdu, 611731 P. R. China

**Keywords:** Complex networks, Statistical physics

## Abstract

The identification of vital nodes that maintain the network connectivity is a long-standing challenge in network science. In this paper, we propose a so-called reverse greedy method where the least important nodes are preferentially chosen to make the size of the largest component in the corresponding induced subgraph as small as possible. Accordingly, the nodes being chosen later are more important in maintaining the connectivity. Empirical analyses on eighteen real networks show that the reverse greedy method performs remarkably better than well-known state-of-the-art methods.

## Introduction

Network science is playing an increasingly significant role in many domains including physics, sociology, engineering, biology, management, and so on^1^. Because of the heterogeneous nature of real networks^2^, the overall connectivity of networks may depend on a small set of nodes, usually named as hub nodes. Taking the Internet as an example, several vital nodes attacked deliberately may lead to the collapse of the whole network^3^. Therefore, an efficient algorithm to identify vital nodes that have critical impacts on the network connectivity can help to better prevent catastrophic outages in power grids or the Internet^3–6^, maintain the connectivity or design efficient attacking strategies for communication networks^7^, improve urban transportation capacity with low cost^8^, enhance robustness of financial networks^9^, and so on.

Till far, to identify vital nodes for network connectivity, the majority of known methods only make use of the structural information^10^. Typical representatives include degree centrality (DC)^11^, H-index^12^, k-shell index (KS)^13^, PageRank (PR)^14^, LeaderRank^15^, closeness centrality (CC)^16^, betweenness centrality (BC)^17^, and so on. Recently, Morone and Makse^18^ proposed a novel index called collective influence (CI), which is based on the site percolation theory and can find out the minimal set of nodes that are crucial for the global connectivity. CI performs remarkably better than some known methods in identifying the nodes’ importance for network connectivity^18,19^. Furthermore, some sophisticated methods with even better performance have been reported, such as the belief propagation-guided decimation method^20,21^ (BPD), the two-core based algorithm^22^ (CoreHD) and the explosive immunization method^23^ (EI).

This paper proposes a novel method named reverse greedy (RG) method. The first word stands for the process that we add nodes one by one to an empty network, which is inverse to the usual process that removes nodes from the original network. The second word emphasizes that we choose the nodes added by minimizing the size of the largest component. Empirical analyses on eighteen real networks show that RG performs remarkably better than well-known state-of-the-art methods.

## Algorithms

**Figure 1:**
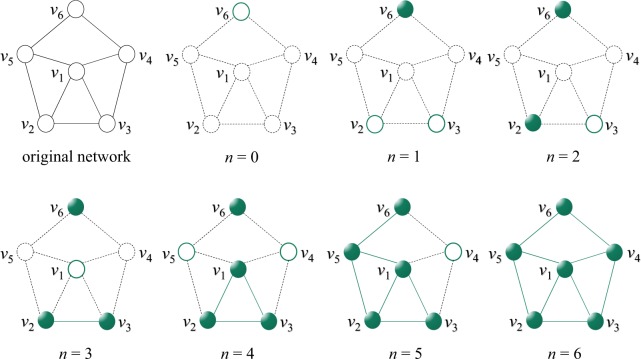
The process of RG in a network with six nodes. Here we use degree as the feature $f$ and for convenience in the later description we set $\epsilon =0.01$ (note that, $\epsilon$ should be positive yet small enough and it is set to be $1{0}^{-7}$ in the later experiments). Initially, the network is empty. At the first time step, ${G}_{1}^{\max }({v}_{1})={G}_{1}^{\max }({v}_{2})={G}_{1}^{\max }({v}_{3})={G}_{1}^{\max }({v}_{4})={G}_{1}^{\max }({v}_{5})={G}_{1}^{\max }({v}_{6})\ =\ 1$, and $cost({v}_{1},1)=1.04$, $cost({v}_{2},1)=1.03$, $cost({v}_{3},1)=1.03$, $cost({v}_{4},1)=1.03$, $cost({v}_{5},1)=1.03$, $cost({v}_{6},1)=1.02$. Therefore, we add node ${v}_{6}$ into the network because $cost({v}_{6},1)$ is the smallest. In the second time step, ${G}_{2}^{\max }({v}_{1})={G}_{2}^{\max }({v}_{2})={G}_{2}^{\max }({v}_{3})=1$, and ${G}_{2}^{\max }({v}_{4})={G}_{2}^{\max }({v}_{5})=2$, so we only compare three candidates ${v}_{1}$, ${v}_{2}$ and ${v}_{3}$. Since $cost({v}_{1},1)=1.04$, $cost({v}_{2},1)=1.03$ and $cost({v}_{3},1)=1.03$, we randomly select a node from $\{{v}_{2},{v}_{3}\}$. Here we choose ${v}_{2}$ for example. Repeat this process until all nodes are added into the network. Finally, we get the ranking of nodes as $\{{v}_{4},{v}_{5},{v}_{1},{v}_{3},{v}_{2},{v}_{6}\}$, in an inverse order of the additions. The symbol $n$ in the bottom of each plot stands for the corresponding time step.

The core of the RG algorithm is the reverse process, which adds nodes one by one to an empty network while minimizes the cost function until all nodes in the considered network are added. Then, nodes are ranked inverse to the order of additions, that is to say, the later added nodes are more important in maintaining the network connectivity. Denote $G(V,E)$ the original network under consideration, where $V$ and $E$ are the sets of nodes and edges, respectively. This paper focuses on simple networks, where the weights and directions of edges are ignored, and the self loops are not allowed. The reverse process starts from an empty network ${G}_{0}({V}_{0},{E}_{0})$, where ${V}_{0}=\varnothing$ and ${E}_{0}=\varnothing$. At the $(n+1)$^th^ time step, one node from the remaining set $V-{V}_{n}$ is selected to add into the current network ${G}_{n}({V}_{n},{E}_{n})$ to form a new network of $(n+1)$ nodes, say ${G}_{n+1}({V}_{n+1},{E}_{n+1})$. Note that, all progressive networks ${G}_{n}$ ($n=0,1,2,\cdots \ ,N$, with $N$ being the size of the original network $G$) in the process are induced subgraphs of $G$. For example, ${G}_{n}$ is consisted of all edges in $G$ with both two ends belonging to ${V}_{n}$. According to the greedy strategy, the selected node $i$ should minimize the size of the largest component in ${G}_{n+1}$. If there are multiple nodes satisfying this condition, we will choose the one with the help of another structural feature of the node $i$ in $G$ (e.g., degree, betweenness, and so on). Therefore, the cost function can be defined as

1 $$cost(i,n+1)={G}_{n+1}^{\max }(i)+\varepsilon f(i),$$

where ${G}_{n+1}^{\max }(i)$ is the size of the largest component after adding node $i$ into ${G}_{n}$, $f(i)$ is a certain structural feature of node $i$ in $G$, and $\epsilon$ is a very small positive parameter and has no effect on the results of RG as long as $\epsilon \ast maxf(i) < 1$ is guaranteed. The parameter $\epsilon$ works only when ${G}_{n+1}^{\max }(\bullet )$ are indistinguishable for multiple nodes. At each time step, we add the node minimizing the cost function into the network, and if there are still multiple nodes with the minimum cost, we will select one of them randomly. This process stops after $N$ time steps, namely all nodes are added with ${G}_{N}\equiv G$. An illustration of such process in a small network is shown in Fig. 1.

## Data

In this paper, eighteen real networks from disparate fields are used to test the performance of RG, including four collaboration networks (Jazz, NS, Ca-AstroPh and Ca-CondMat), two communication networks (Email-Univ and Email-EuAll), four social networks (PB, Sex, Facebook and Loc-Gowalla), one transportation network (USAir), one infrastructure network (Power), one citation network (Cit-HepPh), one road network (RoadNet-TX), one web graph (Web-Google) and three autonomous systems graphs (Router, AS-733 and AS-Skitter). Jazz^24^ is a collaboration network of jazz musicians. NS^25^ is a co-authorship network of scientists working on network science. Ca-AstroPh^26^ is a collaboration network of Arxiv Astro Physics category. Ca-CondMat^26^ is a collaboration network of Arxiv Condensed Matter category. Email-Univ^27^ describes email interchanges between users including faculty, researchers, technicians, managers, administrators, and graduate students of the Rovira i Virgili University. Email-EuAll^26^ is an email network of a large European Research Institution. PB^28^ is a network of US political blogs. Sex^29^ is a bipartite network in which nodes are females (sex sellers) and males (sex buyers) and edges between them are established when males write posts indicating sexual encounters with females. Facebook^30^ is a sample of the friendship network of Facebook users. Loc-Gowalla^31^ describes the friendships of Gowalla users. USAir^32^ is the US air transportation network. Power^33^ is a power grid of the western United States. Cit-HepPh^34^ is a citation network of high energy physics phenomenology. RoadNet-TX^35^ is a road network of Texas. Web-Google^35^ is a web graph of the Google programming contest in 2002. Router^36^ is a symmetrized snapshot of the structure of the Internet at the level of autonomous systems. AS-733^37^ contains the daily instances of autonomous systems from November 8 1997 to January 2 2000. AS-Skitter^37^ describes the autonomous systems from traceroutes run daily in 2005 by Skitter. These networks’ topological features (including the number of nodes, the number of edges, the average degree, the clustering coefficient^33^, the assortative coefficient^38^ and the degree heterogeneity^39^) are shown in Table 1.

**Table 1:** The basic topological features of the eighteen real networks. $N$ and $M$ are the number of nodes and edges, $\langle k\rangle$ is the average degree, $C$ is the clustering coefficient, $r$ is the assortative coefficient and $H$ is the degree heterogeneity.

Networks	*N*	*M*	$\langle {\boldsymbol{k}}\rangle$	*C*	*r*	*H*
Jazz	198	2742	27.6970	0.6334	0.0202	1.3951
NS	379	914	4.8232	0.7981	$-0.0817$	1.6630
Ca-AstroPh	18771	198050	21.1017	0.6769	0.2051	3.0981
Ca-CondMat	23133	93439	8.0784	0.7058	0.1340	2.7305
Email-Univ	1133	5451	9.6222	0.2540	0.0782	1.9421
Email-EuAll	265214	364481	2.7486	0.4532	$-0.1781$	195.3005
PB	1222	16714	27.3552	0.3600	$-0.2213$	2.9707
Sex	15810	38540	4.8754	0	$-0.1145$	5.8276
Facebook	63731	817090	25.6418	0.2532	0.1769	3.4331
Loc-Gowalla	196591	950327	9.6681	0.3163	$-0.0293$	31.7105
USAir	332	2126	12.8072	0.7494	$-0.2079$	3.4639
Power	4941	6594	2.6691	0.1065	0.0035	1.4504
Cit-HepPh	34546	420877	24.3662	0.2962	$-0.0063$	2.6055
RoadNet-TX	1379917	1921660	2.7852	0.0575	0.1304	1.1303
Web-Google	875713	4322051	9.8709	0.6235	$-0.0551$	17.1517
Router	5022	6258	2.4922	0.0329	$-0.1384$	5.5031
AS-733	6474	12572	3.8838	0.3992	$-0.1818$	42.4336
AS-Skitter	1696415	11095298	13.0809	0.2963	$-0.0814$	110.4670

Besides real-world networks, we also work on ErdŐs-Rényi (ER) random networks^40^ with different densities. We generate four ER random networks with $N=100000$ and $\langle k\rangle =6,9,12,15$, named as ER-6, ER-9, ER-12, ER-15, respectively.

## Results

We apply two widely used metrics to evaluate algorithms’ performance. One is called *robustness* $R$^41^. Given a network, we remove one node at each time step and calculate the size of the largest component of the remaining network until the remaining network is empty. The *robustness*$R$ is defined as^41^

2 $$R=\frac{1}{N}\mathop{\sum }\limits_{Q=1}^{N}S(Q),$$

where $S(Q)$ is the number of nodes in the largest component divided by $N$ after removing $Q$ nodes. The normalization factor 1/$N$ ensures that the values of $R$ of networks with different sizes can be compared. Obviously, a smaller $R$ means a quicker collapse and thus a better performance. Another metric is the number of nodes to be removed to make the size of the largest component in the remaining network being no more than $0.01N$, denoted by, ${\rho }_{min}$. Obviously, a smaller ${\rho }_{min}$ means a better performance.

Here we use BC, CC, DC, H-index, KS, PR, CI, CI with reinsertion (short for CI+), BPD, CoreHD and EI as the benchmark algorithms (see details about these benchmark algorithms in Methods). Tables 2 and 3 compare $R$ and ${\rho }_{min}$ of RG and other benchmarks algorithms, respectively. Notice that, we use the random removal (Random) as the background benchmark in order to show the improvement by each method. Both BPD and CoreHD need a refinement process to insert back some removed nodes. In each step, it calculates the increase of the component size after the insertion of a node and select the node that gives the smallest increase. Such process stops when the largest component reaches $0.01N$. These two methods do not provide a ranking of all nodes, and thus we cannot obtain $R$ for them. For EI, according to^23^, we set $K=6$ and the number of candidates being equal to 2000. So the networks with sizes smaller than 2000 fail to apply EI (those cases are marked by N/A in Tables 2–4). Each result of EI is obtained by averaging over 20 independent realizations. The radii of CI and CI+ are set to be 3 only except for AS-Skitter (its radius is set to be 2) because its size is too large and thus $radius=3$ will leads to too much computation.

**Table 2:** The Robustness $R$ for RG and other benchmarks. The best performed method for each network is emphasized in bold.

Networks	Random	BC	CC	DC	H-index	KS	PR	CI	CI+	EI	RG
Jazz	0.4770	0.3956	0.4198	0.4409	0.4497	0.4571	0.4262	0.4598	**0.3381**	N/A	0.3477
NS	0.3453	0.0488	0.1336	0.0540	0.1155	0.1582	0.0524	0.0622	0.0429	N/A	**0.0251**
Ca-AstroPh	0.4332	0.1926	0.2999	0.2526	0.2905	0.3067	0.2146	0.1970	0.1566	0.1579	**0.1477**
Ca-CondMat	0.3927	0.1134	0.2091	0.1261	0.1851	0.2360	0.1062	0.1019	0.0821	0.0774	**0.0727**
Email-Univ	0.4514	0.2578	0.2893	0.2519	0.2836	0.2937	0.2394	0.2271	0.2032	N/A	**0.1844**
Email-EuAll	0.3031	0.0011	0.0084	0.0009	0.0095	0.0095	0.0009	0.0056	0.0056	0.0019	**0.0009**
PB	0.4725	0.2192	0.2908	0.2286	0.2578	0.2611	0.2155	0.3614	0.1909	N/A	**0.1740**
Sex	0.3878	0.0841	0.2208	0.0725	0.0981	0.1142	0.0690	0.0628	0.0579	0.0645	**0.0513**
Facebook	0.4556	0.2935	0.3570	0.3137	0.3328	0.3389	0.2893	0.2674	**0.2311**	0.2371	0.2372
Loc-Gowalla	0.4118	0.1603	0.2545	0.1329	0.1559	0.1673	0.1240	0.1093	**0.0863**	0.0916	0.0896
USAir	0.4168	0.1129	0.1442	0.1228	0.1498	0.1588	0.1072	0.4335	0.1269	N/A	**0.0942**
Power	0.2059	0.0656	0.1973	0.0634	0.1090	0.2628	0.0594	0.0434	0.0417	0.0112	**0.0088**
Cit-HepPh	0.4777	0.3504	0.4259	0.3664	0.3931	0.4022	0.3371	0.3017	**0.2624**	0.2860	0.2657
RoadNet-TX	0.1797	0.0132	0.2714	0.0859	0.1514	0.2052	0.1530	0.0828	0.0323	**0.0019**	0.0028
Web-Google	0.3980	0.1028	0.2739	0.1141	0.2172	0.2575	0.0809	0.0730	0.0541	**0.0322**	0.0379
Router	0.3159	0.0142	0.0686	0.0121	0.0136	0.0276	0.0136	0.0162	0.0206	0.0072	**0.0063**
AS-733	0.4112	0.0142	0.1508	0.0126	0.0246	0.0390	0.0127	0.0138	0.0138	0.0098	**0.0097**
AS-Skitter	0.4395	0.1098	0.2490	0.0706	0.1449	0.1682	0.0527	0.0510	0.0400	**0.0287**	0.0294
ER-6	0.4555	0.3751	0.4110	0.3474	0.4038	0.4200	0.3140	0.2880	**0.2581**	0.2701	0.2697
ER-9	0.4795	0.4340	0.4543	0.4208	0.4557	0.4652	0.3892	0.3618	**0.3285**	0.3423	0.3455
ER-12	0.4886	0.4601	0.4728	0.4534	0.4762	0.4809	0.4306	0.4024	**0.3697**	0.3834	0.3886
ER-15	0.4927	0.4742	0.4826	0.4705	0.4856	0.4881	0.4545	0.4265	**0.3957**	0.4108	0.4147

**Table 3:** The minimum number of nodes ${\rho }_{min}$ for RG and other benchmarks. The best performed method for each network is emphasized in bold.

Networks	Random	BC	CC	DC	H-index	KS	PR	CI	CI+	BPD	CoreHD	EI	RG
Jazz	192	196	196	189	190	191	185	194	165	160	**143**	N/A	160
NS	362	354	370	196	297	330	213	353	221	**60**	165	N/A	109
Ca-AstroPh	17850	6714	13970	8724	11003	11589	7985	5385	4813	4198	6836	4320.60	**4060**
Ca-CondMat	20969	5488	11903	5837	9356	11210	4599	3829	3484	2583	2600	2700.80	**2538**
Email-Univ	937	619	844	540	603	652	501	918	608	**367**	513	N/A	369
Email-EuAll	235019	1791	13479	1431	31634	31515	1456	1163	1193	1064	1070	6985.80	**1053**
PB	1186	541	839	549	708	707	543	1207	608	**378**	388	N/A	379
Sex	13794	3270	10216	2639	3742	4896	2372	1581	1577	**1394**	4505	1896.60	1432
Facebook	51972	37893	47718	34405	38252	40071	32809	23686	23612	22326	36716	22657.00	**22289**
Loc-Gowalla	160823	76000	153855	53828	66860	77534	51158	32441	31905	26696	27148	26916.70	**26187**
USAir	327	179	321	207	273	273	182	315	168	**98**	120	N/A	99
Power	4732	1733	4863	975	3203	3284	1254	625	528	320	327	337.10	**293**
Cit-HepPh	33311	22165	30496	22533	25359	26575	20256	14539	14133	13454	13523	14498.90	**13229**
RoadNet-TX	432809	400581	1351970	307413	704789	1058127	403859	140777	92977	20676	20289	**16800.10**	20158
Web-Google	778750	218764	524970	256099	364054	429372	192520	105764	85418	50878	51603	**41948.85**	47369
Router	4203	442	3419	222	867	765	274	1451	1005	**100**	548	182.00	131
AS-733	4140	298	3682	244	1039	1122	275	194	207	161	156	169.35	**153**
AS-Skitter	1603915	580456	877400	322128	530287	601616	297322	167132	153596	73229	73601	70901.00	**69122**
ER-6	83154	60565	70976	53943	66180	71673	48443	36879	36969	**33571**	45470	36692.70	35641
ER-9	88664	72021	79382	67937	77701	83711	59861	48746	48704	**45670**	51204	49297.85	47836
ER-12	91170	78201	84700	76144	84765	87766	69196	56937	56899	53902	**53586**	57428.10	55961
ER-15	92802	83334	87855	81623	88051	90873	74828	62514	62595	**59837**	62126	63861.00	61725

**Table 4:** Running time of the twelve methods (seconds).

Networks	BC	CC	DC	H-index	KS	PR	CI	CI+	BPD	CoreHD	EI	RG
Jazz	0.01	0.01	0.00	0.01	0.01	0.01	0.34	0.35	0.01	0.01	N/A	0.01
NS	0.01	0.01	0.01	0.02	0.02	0.01	0.08	0.11	0.01	0.01	N/A	0.02
Ca-AstroPh	12.54	3.63	1.07	1.64	0.35	0.26	126.73	136.85	39.54	0.62	652.14	26.77
Ca-CondMat	18.51	6.87	0.27	2.30	0.39	0.26	103.22	128.70	37.11	0.57	604.54	8.28
Email-Univ	0.05	0.03	0.02	0.03	0.02	0.01	1.30	1.43	1.02	0.01	N/A	0.04
Email-EuAll	7466.61	2019.01	2.15	22.32	4.46	1.04	6247.12	11504.13	167.24	0.64	7492.87	407.35
PB	0.10	0.04	0.02	0.04	0.03	0.02	31.75	32.54	1.37	0.01	N/A	0.06
Sex	7.58	3.05	0.11	1.03	0.22	0.11	20.24	20.44	11.32	0.29	39.40	2.27
Facebook	572.50	155.24	1.00	13.26	1.63	1.03	596.30	621.45	54.15	1.26	368.62	105.48
Loc-Gowalla	3920.06	757.08	1.72	25.71	8.78	4.94	5214.30	5366.60	601.55	1.53	4167.43	1076.40
USAir	0.01	0.01	0.00	0.01	0.01	0.00	0.12	0.14	0.16	0.01	N/A	0.01
Power	0.48	0.25	0.04	0.16	0.07	0.04	1.10	1.90	1.30	0.01	11.35	0.29
Cit-HepPh	133.73	34.86	0.58	2.85	0.81	0.56	3126.00	3224.00	394.21	0.37	764.61	30.38
RoadNet-TX	104650.98	45407.24	13.14	516.77	122.47	11.90	400.13	392.70	416.30	5.08	185563.10	4704.60
Web-Google	220440.15	81370.22	9.75	121.26	20.71	10.68	64916.31	62752.74	2754.60	12.33	82456.80	2414.70
Router	0.43	0.24	0.02	0.14	0.05	0.02	0.19	0.35	1.14	0.01	8.91	0.20
AS-733	1.24	0.63	0.07	0.38	0.14	0.05	6.24	7.18	11.59	0.39	132.69	0.22
AS-Skitter	234360.35	64492.47	21.82	462.47	47.86	21.53	21632.00	22549.00	4421.00	37.66	253960.22	11079.00
ER-6	1621.70	162.12	1.42	19.71	1.51	0.97	2683.20	2977.55	157.58	54.89	830.21	327.96
ER-9	1757.48	192.58	1.48	20.25	1.55	1.11	2977.56	3511.60	190.44	65.04	778.32	402.40
ER-12	1812.76	203.38	1.52	20.19	1.64	1.15	3263.45	3749.20	210.19	87.50	770.77	438.60
ER-15	1931.24	221.91	1.62	21.33	1.62	1.24	3691.70	4061.80	246.10	88.30	763.31	468.50

**Figure 2:**
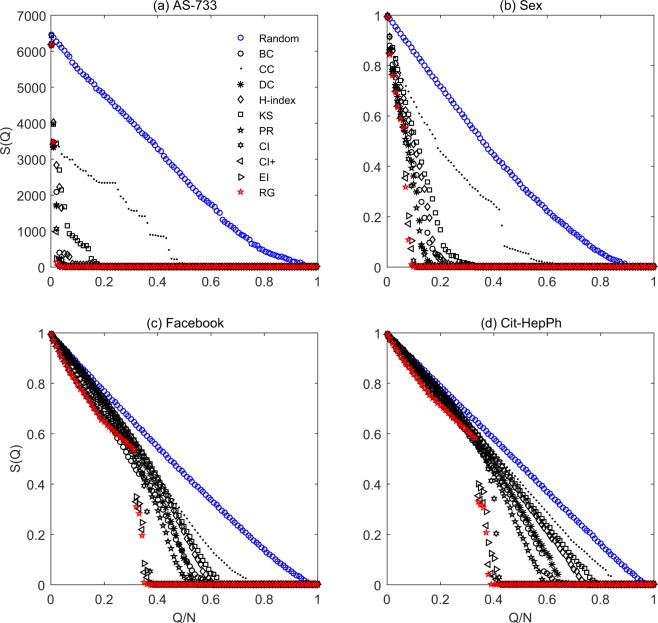
Comparing the performance of the background benchmark (random removal, denoted by blue circles), RG (red stars) and the other benchmark algorithms (black symbols). The $x$-axis is the fraction of nodes being removed (i.e., $Q$/$N$), and the $Y$-axis denotes the number of nodes in the largest component divided by $N$ (i.e., $S(Q)$). The four selected networks are (**a**) AS-733, (**b**) Sex, (**c**) Facebook, and (**d**) Cit-HepPh, respectively. Other networks exhibit similar results.

As shown in Table 2, subject to $R$, CI, CI+, EI and RG perform better than the classical centralities (e.g., BC, CC, DC, H-index, KS and PR) in almost all networks, and RG is overall the best method subject to $R$ for real networks. Figure 2 shows the collapsing processes of four representative networks, resulted from the node removal by RG and other benchmark algorithms. Obviously, RG can lead to faster collapse than all other algorithms, and CI+ is the second best algorithm. As shown in Table 3, subject to ${\rho }_{min}$, BPD, CoreHD, EI and RG perform better than the classical centralities and CI/CI+ in almost all networks. For real networks, RG and BPD perform remarkably better than other methods. For random networks, RG is among the best and much better than the classical centralities. However, RG is slightly worse than CI+ subject to $R$ and BPD subject to ${\rho }_{min}$. In random networks, the topological difference among different nodes is relatively small, and thus RG may mistakenly select some influential nodes as unimportant nodes at the early stage, leading to unsatisfactory results. Table 4 compares the CPU times of the 12 methods under consideration, from which one can see that RG is slower than DC, H-index, KS, PR and CoreHD, similar to CC and BPD and faster than BC, CI/CI+ and EI. Generally speaking, RG is an efficient method.

## Discussion

To our knowledge, most previous methods directly identify the critical nodes by looking at the effects due to their removal^10^. In contrast, our method tries to firstly find out the least important nodes, so that the remaining ones are those critical nodes. To our surprise, such a simple idea eventually results in an efficient algorithm that outperforms many well-known benchmark algorithms. Beyond the percolation process considered in this paper, the reverse method provides a novel angle of view that may find successful applications in some other network-based optimization problems related to certain rankings of nodes or edges.

The performance of RG can be further improved by introducing some sophisticated skills. For example, instead of degree, $f(i)$ can be different for different networks or set in a more complicated way to improve the algorithm’s performance. In addition, the simple adoption of the greedy strategy may bring us to some local optimums. Such shortage can be to some extent overcame by applying the beam search^42^, which searches for the best set of $m$ nodes adding to the network that optimizes the cost function. The present algorithm is the special case for $m=1$. Although beam search is still a kind of greedy strategy, it usually performs much better when $m$ is larger. At the same time, the beam search with large $m$ costs a lot on time and space. Therefore, how to find a good tradeoff is also an open challenge in real practice.

## Methods

### Benchmark centralities

DC^11^ of node $i$ is defined as

3 $$DC(i)=\sum _{j}{a}_{ij},$$

where $A=\{{a}_{ij}\}$ is the adjacency matrix, that is, ${a}_{ij}$ = 1 if $i$ and $j$ are directly connected and 0 otherwise.

H-index^12^ of node $i$, denoted by $H(i)$, is defined as the maximal integer satisfying that there are at least $H(i)$ neighbors of node $i$ whose degrees are all no less than $H(i)$. Such index is an extension of the famous H-index in scientific evaluation^43^ to network analysis.

KS^13^ is implemented by the following steps: Firstly, remove all nodes with degree one, and keep deleting the existing nodes until all nodes’ degrees are larger than one. All of these removed nodes are assigned 1-shell. Then recursively remove the nodes with degree no larger than two and set them to 2-shell. This procedure continues until all higher-layer shells have been identified and all network nodes have been removed.

PR^14^ of node $i$ is defined as the solution of the equations

4 $$P{R}_{i}(t)=s\mathop{\sum }\limits_{j=1}^{N}{a}_{ji}\frac{P{R}_{j}(t-1)}{{k}_{j}}+(1-s)\frac{1}{N},$$

where ${k}_{j}$ is the degree of node $j$ and $s$ is a free parameter controlling the probability of a random jump. In this paper, $s$ is set to $0.85$.

CC^15^ of node $i$ is defined as

5 $$CC(i)=\frac{N-1}{{\sum }_{j\ne i}{d}_{ij}},$$

where ${d}_{ij}$ is the shortest distance between nodes $i$ and $j$.

BC^16^ of node $i$ is defined as

6 $$BC(i)=\sum _{s\ne i,s\ne t,i\ne t}\frac{{g}_{st}(i)}{{g}_{st}},$$

where ${g}_{st}$ is the number of shortest paths between nodes $s$ and $t$, and ${g}_{st}(i)$ is the number of shortest paths between nodes $s$ and $t$ that pass through node $i$.

CI^18^ of node $i$ is defined as

7 $$CI(i)=({k}_{i}-1)\sum _{j\in \partial ball(i,\ell )}({k}_{j}-1),$$

where $ball(i,\ell )$ is the set of nodes inside a ball of radius $\ell$, consisted of all nodes with distances no more than $\ell$ from node $i$, and $\partial ball(i,\ell )$ is the frontier of this ball. CI+ is the version of CI with reinsertion.

BPD^21^ is rooted in the spin glass model for the feedback vertex set problem^44^. At time $t$ of the iteration process, the algorithm estimates the probability ${q}_{i}^{0}(t)$ that every node $i$ of the remaining network $G(t)$ is suitable to be deleted. The explicit formula for this probability is

8 $${q}_{i}^{0}=\frac{1}{1+{e}^{x}\left[1+{\sum }_{k\in \partial i(t)}\frac{1-{q}_{k\to i}^{0}}{{q}_{k\to i}^{0}+{q}_{k\to i}^{k}}\right]{\prod }_{j\in \partial i(t)}[{q}_{j\to i}^{0}+{q}_{j\to i}^{j}]},$$

where $x$ is an adjustable reweighting parameter, and $\partial i(t)$ denotes node $i$’s set of neighbors at time $t$. The quantity ${q}_{i\to j}^{0}(t)$ is the probability that the neighboring node $j$ is suitable to be deleted if node $i$ is absent from the network $G(t)$, while ${q}_{i\to j}^{j}(t)$ is the probability that this node $j$ is suitable to be the root node of a tree component in the absence of node $i$. The node with the highest probability of being suitable for deletion is deleted from network $G(t)$ along with all its associated edges. This node deletion process stops after all the loops in the network have been destroyed. Then check the size of each tree component in the remaining network. If a tree component is too large (which occurs only rarely), an appropriately chosen node from this tree to achieve a maximal decrease in the tree size is deleted. Repeat this node deletion process until all the tree components are sufficiently small.

CoreHD^22^ simply removes highest-degrees nodes from the 2-core in an adaptive way (updating node degree as the 2-core shrinks), until the remaining network becomes a forest. Furthermore, CoreHD breaks the trees into small components. In case the original network has many small loops, a refined dismantling set is obtained after a reinsertion of nodes that do not increase (much) the size of the largest component.

EI^23^ selects $m$ candidate nodes from the set of absent nodes at each step and calculate the score ${\sigma }_{i}$ of each of them using the following kernel

9 $${\sigma }_{i}=\sum _{j\in {N}_{i}}(\sqrt{| | {C}_{j}| | }-1)+{k}_{i}^{(eff)},$$

where ${N}_{i}$ represents the set of all connected components linked to node $i$, each of which has a size ${C}_{j}$, and ${k}_{i}^{(eff)}$ is an effective degree attributed to each node (please see^23^ for the details). Then the nodes with the lowest scores are added to the network. This procedure is continued until the size of the giant connected component exceeds a predefined threshold. The minus one term in Eq. (9) is used to exclude any leaves connected to node $i$, since they do not contribute to the formation of the giant connected component and should be ignored.

## Data Availability

All relevant data are available at https://github.com/ChinaYiqun/network-data.
